# The effect of topiramate on cognitive fMRI

**DOI:** 10.1016/j.eplepsyres.2012.12.007

**Published:** 2013-07

**Authors:** Clarissa Lin Yasuda, Maria Centeno, Christian Vollmar, Jason Stretton, Mark Symms, Fernando Cendes, Mitul A. Mehta, Pamela Thompson, John S. Duncan, Matthias J. Koepp

**Affiliations:** a Institute of Neurology, University College of London, London, UK; b Department of Neurology, University of Campinas, Campinas, Brazil; c Department of Neuroimaging, King's College London, London, UK

**Keywords:** Topiramate, Default mode network, Epilepsy, Language

## Abstract

**Purpose:**

Topiramate (TPM) is known to cause language impairment in healthy volunteers and patients with epilepsy. We assessed the effects of TPM on functional language networks in both patients with focal epilepsies and healthy controls using functional magnetic resonance imaging (fMRI).

**Methods:**

We obtained fMRI data in 24 controls and 35 patients with frontal lobe epilepsy using a simple verbal fluency (VF) paradigm. Eight of the 35 patients were treated with TPM in polytherapy. We compared cognitive task related activations and de-activations in patients taking TPM with patients taking other AEDs and healthy controls. In a longitudinal pilot study with VF-fMRI paradigm, we studied two patients with focal epilepsies twice, prior to starting and on stable doses of TPM, two patients twice, before and after tapering TPM completely and two healthy controls twice, before and after single doses of 200 mg TPM.

**Key findings:**

Cross sectional analyses of VF-fMRI showed a reduction in the task-related deactivation of the default mode network (DMN) in patients taking TPM. The longitudinal study corroborated these findings as both chronic administration and a single dose of TPM were associated with impaired categorical verbal fluency and disruption of task-related deactivations.

**Significance:**

Similar neuropsychological and fMRI findings in patients and healthy controls indicate a specific effect of TPM in default mode network areas that may be essential components of the language network. Our preliminary data suggest a mechanism by which TPM impairs cognitive processing during language function and highlights the sensitivity of fMRI to detect the effects of AEDs on cognitive brain networks.

## Introduction

A major concern when selecting appropriate anti-epileptic drug (AED) treatment is adverse effects on cognition and behavior (Thompson et al., 2000; Loring et al., 2011). Expressive language difficulties, particularly word-finding and dysnomia as well as working memory impairment (Lee et al., 2003; Szaflarski and Allendorfer, 2012), are rather specific for topiramate (TPM), and rarely seen with other AED. These effects have been described not only in patients with epilepsy, but also in people with migraine (De et al., 2008). However, the mechanisms involved are poorly understood and there is no clinical test available that reliably predicts who is at risk of developing side-effects (Brandes et al., 2004; Loring et al., 2011). FMRI revealed decreased activation of prefrontal cortex in response to a verbal task in TPM-treated epilepsy patients (Jansen et al., 2006; Szaflarski and Allendorfer, 2012) while withdrawal of TPM was associated with improved cognitive measures sensitive to frontal lobe functioning (Kockelmann et al., 2003). A recent study demonstrated a dose-related effect of TPM on language fMRI, affecting areas of resting state network (Szaflarski and Allendorfer, 2012) in patients with TLE.

In this study we aimed to investigate regional effects of TPM on cognitive fMRI activation patterns and used two approaches. First, we analyzed fMRI studies in a cohort of frontal lobe epilepsy (FLE) patients with regards to AEDs taken; second, in a prospective, open-label pilot study, we compared cognitive task related activations and de-activations in two healthy controls who received single doses of 200 mg TPM, and four patients who either started or stopped TPM for clinical reasons.

## Methods

For the cross-sectional study we obtained verbal fluency (VF) fMRI and neuropsychological data of 35 patients (15 females, age: median 33 – interquartile range [IQR]: 18 years) with either cryptogenic frontal lobe epilepsy (FLE) or FLE due to focal cortical dysplasia (17 right-sided frontal focus, 13 left-sided and 5 uncertain frontal lobe lateralization). Patients with large lesions were excluded from this study. Patients were recruited from the epilepsy clinics at our center (London, UK) (Vollmar et al., 2011). Twenty-four healthy controls (13 females, age: median 30 – IQR: 7 years) without a history of epilepsy or other neurological condition were included for comparison. Eight of the 35 patients were treated with TPM (dose range: 50–500 mg; median 187.5 mg) given in polytherapy, the remaining 27 patients formed the other-AED group. Clinical data are displayed in Table 1A.

For the longitudinal pilot study, four patients with focal epilepsies (1 temporal lobe epilepsy (TLE) and 3 extra-TLE) were studied as part of their pre-surgical assessment with the same VF-fMRI paradigm (Bonelli et al., 2011). FMRI was acquired in two patients prior to starting TPM (TPM-off), and repeated once patients were taking a working dose (TPM-on), and in two patients before withdrawal (TPM-on) and again after complete cessation (TPM-off). These changes were made for clinical reasons. In addition, two healthy male controls were studied before (TPM-off) and 3 h after a single oral dose of 200 mg TPM (TPM-on).

The study was approved by the Research Ethics Committee of the UCL Institute of Neurology and UCL Hospitals. (Informed consent was obtained from each participant.)

All subjects underwent a brief neuropsychological evaluation of verbal fluency (letter S and category) and backwards Digit Span in close proximity to their fMRI scans (clinical data are displayed in Table 1B). Neuropsychological test performance was compared using Wilcoxon tests.

All fMRI studies were performed on a 3T GE Excite HDx scanner as described previously (Vollmar et al., 2011), with acquisition of gradient-echo planar T2*-weighted images (TE = 25 ms, TR = 2500 ms), providing blood oxygenation level-dependent (BOLD) contrast. The VF-fMRI paradigm consisted of a 5.5 min blocked design with alternating periods of 30 s of a task (subjects requested to covertly generate different words beginning with a visually presented letter, A, S, W, D and E) and baseline (cross-hair fixation) (Bonelli et al., 2011). We analyzed the fMRI data within the framework of the general linear model using SPM5 (http://www.fil.ion.ucl.ac.uk/spm5), including two level random effects analysis. Prior to analysis, the data was realigned and spatially normalized (using a T1-weighted structural image co-registered to the time-series to determine the parameters). The first level model included the task blocks with the baseline coded implicitly and the six movement regressors. The second level was used to test:

1) VF activations and deactivations in each group (TPM, other-AEDs, controls);
2) Changes in BOLD signal associated to different TPM doses.

Activations are reported at a threshold of *p* < 0.05 (corrected for multiple comparisons with false discovery rate method). Deactivations are reported with threshold (*p* < 0.001) uncorrected for multiple comparisons due to the preliminary nature of this work.

1) FMRI data was analyzed with a fixed-effect statistical model to:
2) Identify activation and deactivations for VF for each acquisition (TPM-on/-off);
3) Compare the differences in deactivations between the two acquisitions (TPM-on versus TPM-off) for each subject;
4) To investigate commonalities amongst subjects using a conjunction analysis (Friston et al., 2005).

Resulting maps were thresholded at *p* < 0.001, uncorrected for multiple comparisons.

Patients performed a letter fluency test outside the scanner, in which they were instructed to say as many words as possible starting with the letter “S” in 1 min (Bonelli et al., 2011). For correlation analysis the individual fMRI maps were regressed against the TPM doses. To compare clinical and neuropsychological scores we used Mann–Whitney *U*-test.

## Results

Letter fluency “S” was reduced for patients taking TPM compared to other FLE patients (*p* = 0.053). A single dose of TPM led to a reduction of letter fluency “S” (median reduction 42%, IQR 50%, *p* = 0.078), category fluency (median 26%, IQR 25%, *p* = 0.028) and backwards Digit Span Score (median 14%, IQR 22.5%, *p* = 0.17).

**Figure 1:**
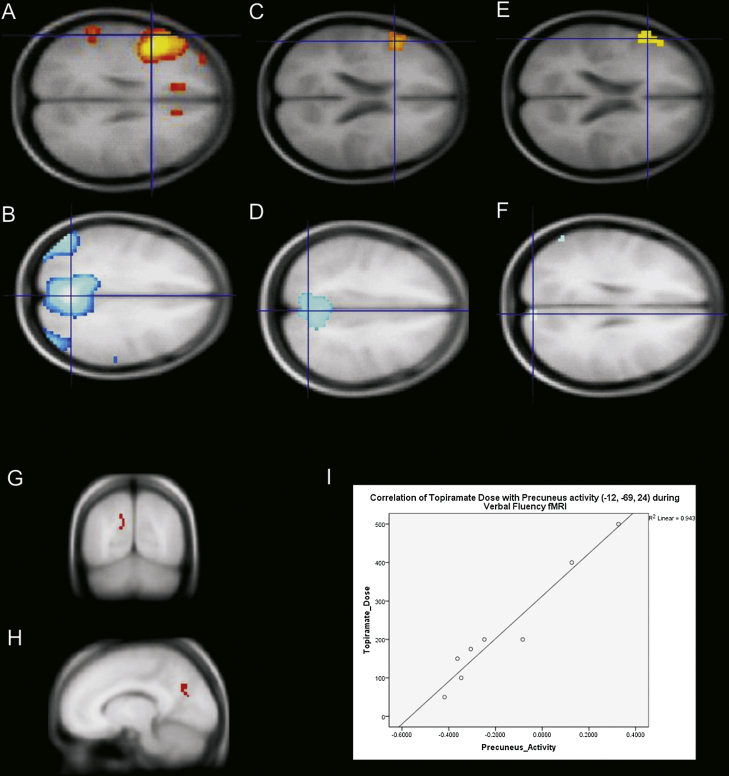
FMRI results (cross-sectional analysis). Red maps represent activations and blue maps represent deactivations. (A and B) controls, (C and D) other FLE patients and (E and F) TPM patients. The reduced area of deactivation in TPM-group (F) suggests a specific effect of this AED on DMN activity. (G) and (H) are correlational analysis, showing the activation in precuneus correlated positively with increasing TPM dosage. (I) Correlation of fMRI activation for verbal fluency and TPM dosages at peak voxel in precuneus.

For the cross-sectional study, group maps showed task-related activations within the left frontal cortex for controls and patients with and without TPM (Fig. 1A, C and E). Conversely, tasks related deactivations were observed in controls and FLE taking other AED, but no significant deactivations were identified in TPM group (Fig. 1D–F). Furthermore, activity in the DMN areas during the task correlated with an increasing dose of TPM (*R* = 0.97) (Fig. 1G–I).

**Figure 2:**
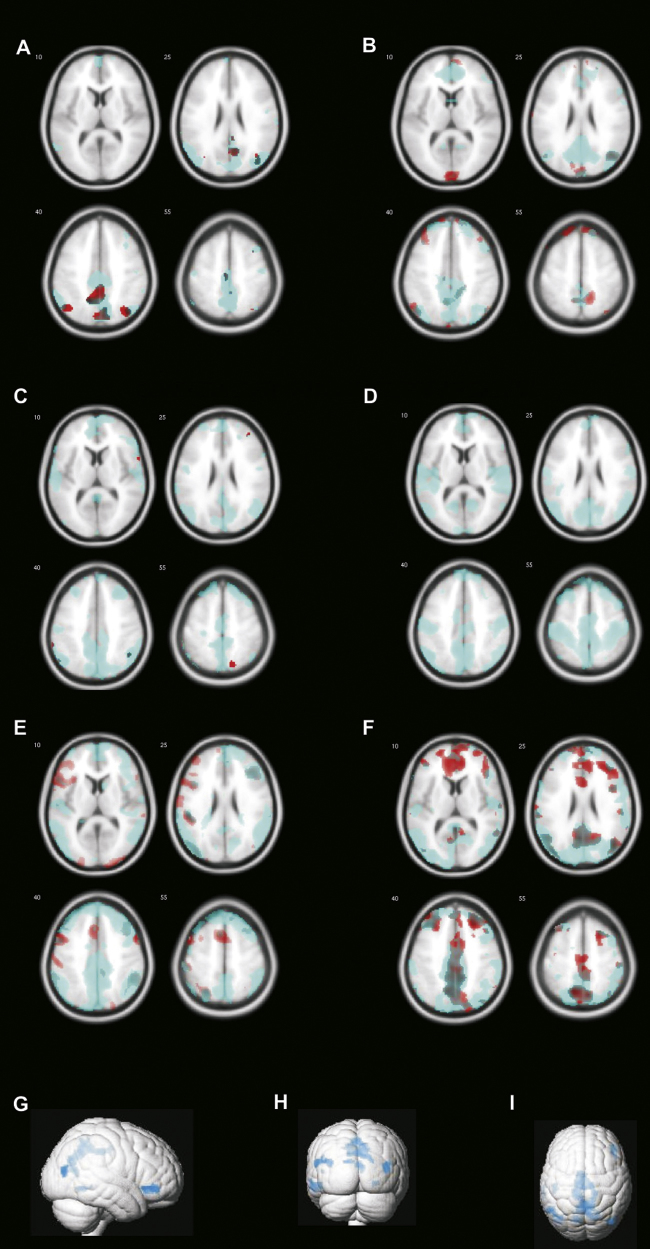
FMRI results (longitudinal pilot study). Red maps represent deactivations from TPM-on status and blue maps represent deactivations from TPM-off. The reduced deactivations in red suggest a specific effect of TPM in the DMN. (A–D) Patients; (E–F) healthy controls. (G) Results from the conjunction analysis, illustrating the common areas from 6 difference maps, including precuneus, bi-parietal and frontal areas.

For the longitudinal pilot study, fMRI analysis showed a non-significant decrease in activation of normal language areas during the TPM-on condition. Task-related deactivations revealed the typical default mode network (DMN) during the TPM-off scans (areas in blue in Fig. 2 (patients A–D, controls E and F), but not during the TPM-on scan (red in Fig. 2). For each individual, the differences between the deactivation maps on–off topiramate were mainly located in areas of the DMN, i.e. precuneus, frontal pole and parietal lobes. Conjunction analysis showed that the common areas of difference across the 6 subjects are within the DMN (Fig. 2G–I).

## Discussion

In subjects taking topiramate, we detected a failure to deactivate the DMN during verbal fluency fMRI, even after a single high dose or during chronic treatment. This impaired deactivation was correlated with an increasing dosage of TPM, indicating a possible specific effect of TPM on cognitive (language) networks. Appropriate deactivation of the DMN during cognitive tasks is necessary for the correct performance. Our findings are in keeping with a recent study of TLE patients showing deactivations in frontal lobes related to increased doses of TPM (Szaflarski and Allendorfer, 2012). In addition, by testing the same subjects with and without TPM effect in a more powerful longitudinal analysis, we could replicate the initial findings from our cross-sectional analysis that the failure to deactivate the DMN was associated with the TPM treatment.

In addition, patients presented with decreased activation of normal language network fMRI in relation to a reduction of neuropsychological language scores, for both TPM and other AEDs, in accordance with previous studies (Szaflarski and Allendorfer, 2012; Jansen et al., 2006).

One possible mechanism directly associated with the dysfunction of DMN relates to the inhibition of carbonic anhydrase by TPM (similarly to acetazolamide), which causes an increased cerebral blood flow without increasing oxygen consumption, resulting in an intensification of the resting bold signal and attenuation of the bold signal during activation (Bruhn et al., 1994).

The dysfunction of DMN and the consequent disruption of language processing are in agreement with the view of the DMN being closely linked to networks of semantic processing and other language functions (Binder et al., 2009). We observed these TPM effects in both temporal and extratemporal lobe patients (TPM add-on) and healthy controls (after a single dose), which provides evidence for a TPM specific effect in default mode areas, regardless of treatment with other AEDs or underlying pathology.

We cannot exclude, though, from this limited data that other AEDs have a similar effect on default mode network, although we did not observe this failure to de-activate for patients treated with carbamazepine or levetiracetam (data not shown).

## Conclusions

Our preliminary data suggest that fMRI may be useful to detect subtle drug effects on cognitive networks. Currently, it remains unclear why some patients develop side-effects on AEDs, and others do not. Beyond the common dose-related adverse effects, there are no reliable predictors of adverse effects of AED treatment. In this context, fMRI investigations could help to stratify patients for specific treatments allowing individualization of treatments and determining where in the brain the effect of AEDs on cognition occurs. Such studies would be applicable to a broad range of CNS diseases with TPM also being an effective or prophylactic treatment for headaches and addiction. Larger longitudinal studies in both patients and controls will be necessary to assess the specific influence of TPM and other anti-epileptic medication on cognitive function.

## Author contributions

CV, MC, MS and PT contributed to data acquisition and data analysis, CY and JS performed statistical analysis, JD and MK were involved in conception and analysis, FC, MM helped with the interpretation and presentation of the data as well as writing of the article.

## Conflicts of interest

Dr Yasuda was supported through FAPESP (09-51425-6/Brazil). Dr Maria Centeno has received funding from Fundacion Caja Madrid. Dr Mitul Mehta reports research funding from Eli Lilly and acts as a consultant for UCB pharma. He is also a scientific advisor for Cambridge Cognition. Drs Christian Vollmar, Jason Stretton, Mark Symms and Pamela Thompson report no disclosures. Prof. Fernando Cendes serves on the editorial boards of Neurology^®^, Epilepsy Research, Epileptic Disorders, and Arquivos de Neuropsiquiatria, and has served on the editorial board of Epilepsia; receives research support from Fundação de Amparo à Pesquisa do Estado de São Paulo (FAPESP) and Conselho Nacional de Pesquisa (CNPq) Brazil; and serves as Chair of the Diagnostic Methods Commission for the International League Against Epilepsy. Prof. John S. Duncan serves on scientific advisory boards for and/or has received funding for travel from GE Healthcare, UCB Pharma, Eisai, Janssen Cilag; and has received honoraria from UCB Pharma and Eisai. Prof. Matthias Koepp served on scientific advisory board of GE, received honoraria from UCB, EISAI and BIAL (Portugal), funding for travel from UCB, Pfizer and Desitin, research support from MRC, Wellcome Trust Foundation and EU-Framework 7 programme, and holds shares in GSK. We confirm that we have read the Journal's position on issues involved in ethical publication and affirm that this report is consistent with those guidelines.

## Figures and Tables

**Table 1:** Clinical data from cross-sectional study (A) are displayed as median (interquartile range); the longitudinal pilot study (B) data shows individual clinical parameters and neuropsychological scores from outside scanner acquired on and off TPM.

(A) Cross-sectional data			
	Controls (24)	TPM (8)	Other AEDS (27)
Age (years)	30 (7)	30 (13)	35 (20)
Gender (f/m)	13/11	4/4	11/16
NART-IQ	111 (10.5)	96 (12)	97 (14)
Letter fluency “S”	15.5 (7)	9 (8)	10 (4)
Categorical fluency	23.5 (4)	17 (10)	18 (9)
Age of onset	–	7.5 (3)	9 (6)
Duration (years)	–	24.5 (14)	21 (16)
Seizure frequency (partial/month)	–	15 (25.5)	20 (116)
Specific AED dose (mg/day)	–	187.5 (175)	–
Polytherapy (%)	–	100%	100%
Number of AEDs	–	3 (1.5)	3 (1)

(B) Longitudinal pilot study						
	Subject 1	Subject 2	Subject 3	Subject 4	Subject 5	Subject 6
Age	35	23	22	40	28	56
Age of onset (years)	6	4	21	9	–	–
Duration (years)	29	19	1	31	–	–
Classification	FLE	Occipital Tuberous	FLE	TLE Temporal	Control	Control
MRI	Normal	Sclerosis	Normal	Resection	Control	Control
Type of intervention	ON–OFF	ON–OFF	OFF–ON	OFF–ON	Acute	Acute
TPM dose (mg)	200	300	150	325	200	200
Serum levels (μmol/L)						
Range 6–74	–	–	–	–	8	0.48

	On	Off	On	Off	On	Off	On	Off	On	Off	On	Off
Letter fluency	8	16	12	22	19	14	3	11	23	37	22	22
Categorical fluency	10	13	16	31	26	35	9	11	13	45	16	22
Digit backwards	–	7	2	4	4	7	4	5	11	14	9	7
Other AEDs (number)	3	3	2	2	–	–	2	3	–	–	–	–
